# Impact of Surface Sealing on Color Stability and Surface Roughness of Conventional Dental Resin Composites

**DOI:** 10.3390/ma18245543

**Published:** 2025-12-10

**Authors:** Georgiana Osiceanu, Sebastian Ciurescu, Liliana Porojan

**Affiliations:** 1 Department of Dental Prostheses Technology (Dental Technology), Center for Advanced Technologies in Dental Prosthodontics, Faculty of Dental Medicine, Doctoral School, Victor Babes University of Medicine and Pharmacy Timisoara, Eftimie Murgu Square No. 2, 300041 Timisoara, Romania; 2 Doctoral School, Victor Babes University of Medicine and Pharmacy Timisoara, Eftimie Murgu Square No. 2, 300041 Timisoara, Romania; 3 Department of Dental Prostheses Technology (Dental Technology), Center for Advanced Technologies in Dental Prosthodontics, Faculty of Dental Medicine, Victor Babes University of Medicine and Pharmacy Timisoara, Eftimie Murgu Square No. 2, 300041 Timisoara, Romania

**Keywords:** dental resin composites, adhesive, sealing, surface roughness, color evaluation, surface treatment

## Abstract

The objective of this study was to evaluate whether any coating material would have a beneficial influence on maintaining color stability and surface roughness and to what extent an uncoated resin composite can keep its original color. The study evaluated three direct composite resins (Gradia Direct Anterior A2, Tetric EvoCeram A2, Filtek Z550 A2) using 30 samples per material (1 mm thick, 14 × 10 × 1 mm). Samples were prepared in 3D-printed molds, light-cured for 40 s, and initially smoothed with abrasive paper (grit 400–2000). The surface treatments applied were as follows: group 1—polished with a brush and Compo + polishing paste, group 2—conditioned with 37% phosphoric acid, ScotchBond adhesive applied, light-cured. All samples were cleaned ultrasonically for 5 min. Initial surface roughness and color were measured with a profilometer and spectrophotometer. Samples were then immersed in distilled water (control at 37 °C), Coca-Cola and red wine (at 10 °C) with surface roughness and color changes measurements taken on days 1, 7, 14 and 90. Immersion media were refreshed weekly. The most notable color changes after immersion in coloring solutions were observed in the groups treated with Coca-Cola and red wine compared with the control group in distilled water. Tetric EvoCeram sealed and Gradia sealed maintained the greatest resistance to perceptible coloration over 90 days, while Filtek Z550 performed the poorest. Tetric EvoCeram sealed maintained the greatest color stability (ΔE < 3.5 at 90 days), whereas Filtek Z550 sealed showed early degradation. Roughness is often decreased by surface sealing. As immersion time rises, unsealed surfaces often become noticeably rougher than sealed ones. This study simulates the oral environment and the exposure of restorative materials to staining agents. As the loss of esthetic properties over time is a continuous problem, the clinical significance of this research lies in demonstrating how a restorative material could resist pigmentation, when in contact with well-known high staining beverages, in order to maintain its esthetic properties and remain suitable for long-term use in the oral cavity. Moreover, the hypothesis that a coating material would protect the resin composite surface and reduce discoloration and surface roughness was tested.

## 1. Introduction

Resin composites are a type of dental material that has been used since the mid-1960s. The composition of this material has four parts: an organic polymer matrix, inorganic filler particles, a silane coupling agent that holds the filler to the matrix, and initiators or inhibitors that control the polymerization process [1]. Apart from this category of materials, there are other restorative options as well, such as amalgam and glass ionomer cement. In an attempt to overcome the disadvantages of mercury-containing amalgams, glass ionomer has been used as a great alternative [2]. Their advantages lie in being economical, ecologically friendly, resistant to decay and capable of promoting remineralized dentine [2].

On the other hand, benefits of resin composites are that they are widely used in restorative dentistry because they offer a variety of shades and opacities, are easy to handle, and are generally more affordable than indirect restorations, but these materials still have some disadvantages that might shorten the life of restorations, such as the fact that they fail to preserve their color well [3,4,5,6]. So, their main problems are their limited color stability, excessive deposits of plaque and marginal leakage caused by the polymerization shrinkage [7,8,9].

Clinical success depends on dental composite materials having acceptable levels of surface roughness, color stability and microhardness as well as the fact that patients’ esthetic expectations have risen, making the color match and long-term color stability of restorative materials increasingly important in recent years [10,11,12].

The degree of polymerization and the kind, size and form of the material’s inorganic filler particles are the main factors that influence the resin composites’ tendency to change color [13]. However, the color modifications may come from both internal and exterior sources not only material-related ones [14]. One significant internal element identified as responsible for the color change in composite resins over extended periods is the oxidation of monomers or catalysts [15]. The patient’s diet, smoking patterns and lack of dental hygiene are examples of external factors that might affect restorative color [15].

The CIELAB system defines color using three parameters: color from a* red (+ a*) to green (−a*), color from b* (yellow + b*) to blue (−b*), and L* brightness between black (0) and white (100) [16,17].

Another essential factor when discussing the aesthetic stability of resin composites is surface roughness, which is indirectly related to the development of periodontal problems and tooth decay, as it promotes plaque accumulation and subsequently gingival irritation, with the literature stating that values greater than 0.2 μm are associated with increased biofilm adherence, and those higher than 0.5 μm are perceived by patients as uncomfortable, thus requiring polishing steps to resolve the issue [18,19,20,21,22,23].

The porosity of the material, the composition of the composite resin, and the polishing methods all affect how rough the surface is [24,25]. In order to fix surface flaws and imperfections, stop microleakage, strengthen the marginal seal, boost wear resistance, and improve stain resistance, alternative materials, such as sealant agents, have been used [9,26,27].

Surface sealants can help resin composite restorations last longer and minimize porosities, microleakage, and wear [28,29]. They can also repair minor restoration defects, reduce surface discoloration, improve the marginal adaptation of resin composites in the posterior area and fix microfissures and other defects caused by polymerization shrinkage and finishing, according to clinical research [28,29,30,31].

In clinical practice, dentists utilize adhesive solutions instead of surface sealants to make the surface of the restorations smoother, which makes the finishing process better [32]. Even though these materials are used over restorations, they may include hydrophilic monomers and solvents that might affect the restorations’ qualities, such as their color stability [32].

There is limited knowledge about the effectiveness of this adhesive system when used as a coating material for promoting the long-term color stability and surface smoothness of resin composite restorations. In the current literature, there are conflicting data regarding the improvement of surface roughness when using a sealant. Some researchers claim that the sealant decreases surface roughness, while others report no significant changes in clinical results over time [33]. Also, the use of different surface sealants (either specially fabricated for this purpose or adhesive systems tested as surface sealants) as a surface treatment to improve surface roughness, color stability after storage in multiple coloring media, and wear has been investigated in numerous studies [10,29,33,34,35,36].

Consequently, the objective of this study was to evaluate the color change and surface roughness of three different resin composites, after immersion at different time points in well-known coloring media, with two surface treatments: finished and coated. The null hypotheses were as follows: H1: There were no significant differences between the finished and sealed surfaces; H2: There were no significant differences in color change with respect to the immersion media and time points; H3: There were no significant differences in surface roughness with respect to the immersion media and time points; H4: There was no correlation between the color change and the surface roughness.

## 2. Materials and Methods

This in vitro experimental study investigated a total of 180 specimens (30 per material group) across five time points: baseline, 1, 7, 14, and 90 days. The study evaluated the extent to which the immersion medium, time, restorative material, and the clinically relevant surface treatment being tested influenced color stability and surface roughness.

### 2.1. Specimens Preparation

Three commonly used direct composite resins (shade A2)—Tetric EvoCeram, Gradia Direct Anterior, and Filtek Z550—were evaluated after being subjected to two surface treatments: polishing alone and polishing followed by a sealant application. Rectangular plate specimens with uniform dimensions (approximately 14 × 10 × 1 mm) were fabricated in a rigid 3D-printed mold that was digitally designed and subsequently printed using the Asiga Max 3D printer (ASIGA Pty Ltd., Alexandria, Australia). They were compacted between glass plates and photopolymerized for 40 s (20 s on each side) through celluloid strips, according to the manufacturers’ recommendations using the Woodpecker LED.B (Guilin Woodpecker Medical Instrument Co., Ltd., Guilin, China) curing light. All specimens then underwent standardized finishing using abrasive papers of increasing grit (400 to 2000). The polished surfaces were further refined with Compo+ polishing paste. For the sealed condition, the prepared surface was etched with 37% orthophosphoric acid for 20 s, thoroughly rinsed for 40 s, air-dried for 10 s, coated with a thin adhesive layer (Scotchbond), air-thinned for 5 s, and light-cured for 30 s to form a continuous surface seal [33]. Immediately before baseline measurements, all specimens were ultrasonically cleaned for 5 min in order to remove debris and residues. The manufacturers and compositions are presented in Table 1.

Each specimen was immersed individually in 30 mL of one of three media: distilled water (control), Coca-Cola Regular (The Coca-Cola Company, Atlanta, GA, USA) and red wine (Domeniile Viticole Tohani, Feteasca Neagra, semi-dry). Storage was at 37 °C for distilled water and 10 °C for Coca-Cola and wine. The temperatures were controlled using a digital thermometer. The immersion solutions were refreshed weekly for the duration of the testing period. Measurements were taken at baseline (immediately after finishing or sealing) and at 1, 7, 14, and 90 days of immersion.

### 2.2. Color Evaluation

Color was recorded with a calibrated spectrophotometer (Vita Easy Shade IV, Zahnfabrik, Bad Säckingen, Germany) in CIEL*a*b* coordinates under D65 standardized illumination and positioning (the maintenance of a 90-degree angle between the probe tip and the sample surface). The calibration of the apparatus was performed before each measurement.

The values were recorded on both white and black background using the WhiBal G7 gray card (White Balance Pocket Card). Color changes were evaluated considering all three color parameters, L*, a*, and b*. The L* coordinate represents lightness with values interpreted as follows: L* = 100 corresponds to absolute white, and L* = 0 corresponds to absolute black. The a* coordinate indicates chromaticity along the red (positive) to green (negative) axis, while the b* coordinate indicates chromaticity along the yellow (positive) to blue (negative) axis.

For each specified time point, ΔE*ab was calculated against the specimen’s own baseline using the Euclidean metric:

(1) $$\Delta E_{ab}=\sqrt{{\left(\Delta L\right)}^{2}+{\left(\Delta a\right)}^{2}+{\left(\Delta b\right)}^{2}}$$

For clinical interpretability, ΔE was also correlated with widely accepted perceptibility ranges: (0 < ΔE < 1 an observer does not notice the difference; 1 < ΔE < 2 only an experienced observer can notice the difference; 2 < ΔE < 3.5 an unexperienced observer also notices the difference; 3.5 < ΔE < 5 a clear difference in color is noticed; 5 < ΔE an observer notices two different colors [37]), which informed categorical summaries, logistic modelling, and the definition of the time-to-event endpoint.

### 2.3. Surface Roughness Evaluation

Surface texture was assessed with contact profilometry, as well as the resulting Ra (μm) (arithmetic mean roughness) and Rz (μm) (maximum vertical surface roughness) values, at the same time points to ensure comparability across materials and finishes. In order to record these parameters, the contact profilometer with a 2 μm stylus, Surftest SJ-201 (Mitutoyo, Kawasaki, Tokyo, Japan) was used. The measurements were taken in three different areas of each sample, and the mean value was calculated. The cut-off value was 0.8 mm, and a force of 0.7 N was applied.

### 2.4. Statistical Analyses

All analyses were performed in JASP v0.95.2. Data integrity was checked through range validation, type consistency, outlier screening, and residual diagnostics for model-based procedures.

The primary outcome was the change in color in CIEL*a*b* space (ΔE*ab) relative to baseline; secondary outcomes included the evolution of L* parameter, the investigation of the changes observed over the testing period in all three environments and the relationship between ΔE and surface roughness parameters. A prespecified binary endpoint, barely visible versus more than barely visible by 90 days, was derived from ΔE thresholds and used for logistic modelling and time-to-event analyses.

Because each specimen contributed repeated measurements, global effects on color difference (ΔE) and luminosity (L*) were assessed with mixed repeated-measures ANOVA. Student’s T tests were employed were needed. The within-subject factor was time (baseline, 1, 7, 14, 90 days); the between-subject factors were immersion medium (water, Cola, red wine) and material/surface group (e.g., Tetric EvoCeram polished, Tetric EvoCeram sealed). Sphericity was evaluated with Mauchly’s test, and we report estimated marginal means with 95% confidence intervals, partial η^2^ as the effect size, and Holm-adjusted post hoc contrasts for pairwise comparisons within significant effects.

To complement the mixed models at clinically relevant time points, between-group differences in ΔE were further examined with one-way ANOVA, which was followed by multiplicity-controlled post hoc tests and standardized effect sizes. The association between color change and roughness (Ra, Rz) was examined at each time point using Pearson correlation with optional partial correlations controlling for immersion medium and material group. Scatterplots with fitted regression lines and 95% confidence bands were generated to visualize relationships.

For the clinical endpoint at 90 days, ΔE was dichotomized at the prespecified threshold of >2, distinguishing “barely perceptible or less” from “more than barely perceptible.” A multivariable logistic regression model was then fit with discoloration status as the dependent variable and the material/surface group, immersion medium, and roughness as predictors. Model performance was summarized by pseudo-R^2^ indices, likelihood-ratio statistics, and discriminatory ability quantified by ROC curves and the area under the curve (AUC). In view of small-sample risks in some groups, penalized (Firth) logistic or exact logistic regression was considered as a sensitivity strategy to address quasi-separation and stabilize estimates.

To characterize the dynamics of discoloration onset, a time-to-event variable was defined as the earliest scheduled day at which the ΔE value exceeded 2. Specimens that remained ≤2 by 90 days were censored at that point. Kaplan–Meier curves were estimated for each material/surface group accompanied by life tables, risk tables, and survival plots to illustrate timing and attrition.

All statistical tests were two-sided with α = 0.05. Multiplicity within families of post hoc contrasts was controlled using Holm’s method. Sensitivity analyses included alternative ΔE thresholds, the exclusion of prespecified outliers, and stratification by immersion medium. No a priori sample-size calculation was performed; instead, equal allocation across materials and media was adopted to maximize balance and power within fabrication constraints.

Because this was an in vitro study using commercially available dental materials and involving no human or animal subjects, ethics approval was not required. All figures (estimated marginal mean trajectories, grouped box/violin plots, correlation scatterplots, ROC curves, and Kaplan–Meier curves with numbers at risk) were generated within JASP’s PlotBuilder to ensure consistent aesthetics and reproducibility.

## 3. Results

A total of 180 specimens (30 per material group) were followed across five time points (baseline, 1, 7, 14, and 90 days). Repeated-measures analyses on luminosity (L*) and color difference (ΔE) demonstrated large, statistically significant effects of time, immersion medium, and material with higher-order interactions showing that color modification trajectories depended on both the liquid and the restorative material.

### 3.1. Luminosity (L*) over Time

**Figure 1:**
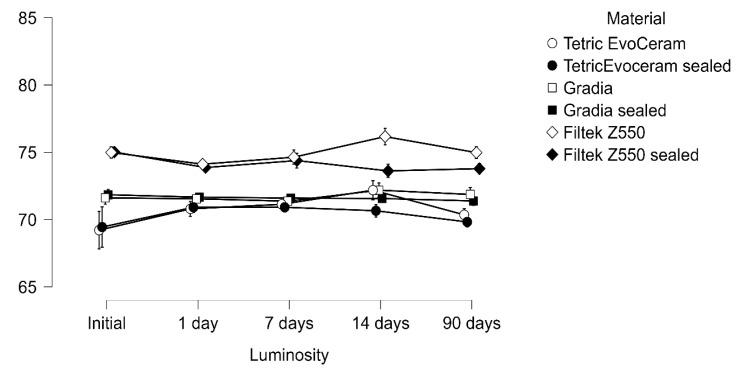
Luminosity measured after immersion in distilled water at 37 °C evaluated at baseline, 1, 7, 14, and 90 days.

**Figure 2:**
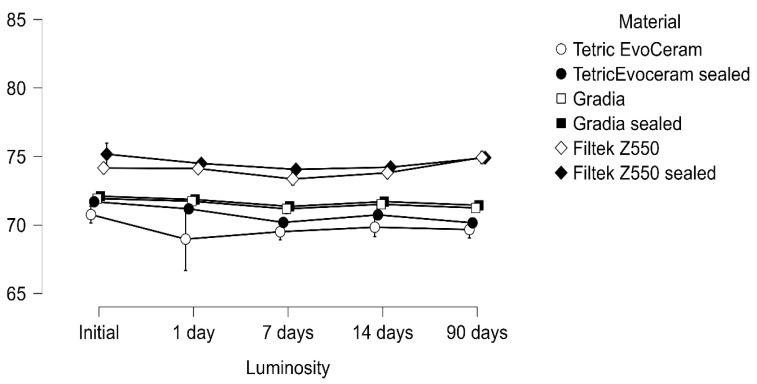
Luminosity measured after immersion in Coca-Cola at 10 °C evaluated at baseline, 1, 7, 14, and 90 days.

**Figure 3:**
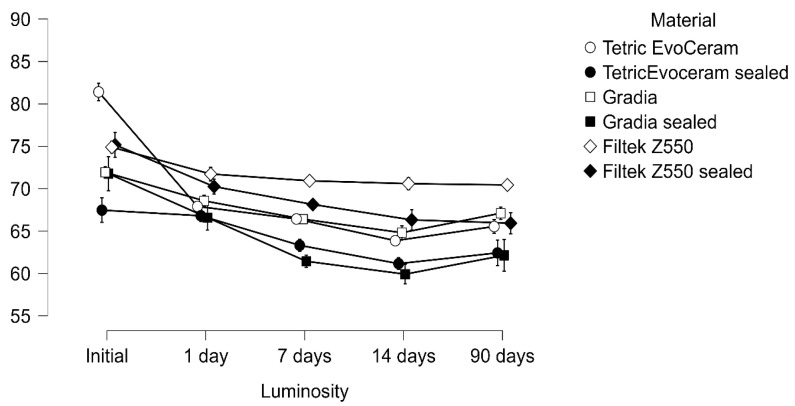
Luminosity measured after immersion in Wine at 10 °C evaluated at baseline, 1, 7, 14, and 90 days.

The repeated-measures ANOVA demonstrated a significant reduction in luminosity over time with strong main effects of immersion, material, and their interaction (all *p* < 0.001). A three-way interaction (luminosity × immersion × material) confirmed that color change trajectories were not uniform but rather depended jointly on both the liquid medium and the restorative material (Table 2 and Table 3; Figure 1, Figure 2 and Figure 3).

Post hoc pairwise contrasts for luminosity showed that values differed significantly between baseline and all follow-up time points with mean differences of 1.86 (Initial vs. 1 day), 2.79 (Initial vs. 7 days), 3.10 (Initial vs. 14 days), and 2.91 (Initial vs. 90 days; all pHolm < 0.001). Additional contrasts among follow-up visits were also significant for 1 day vs. 7, 14, and 90 days (MDs 0.93, 1.24, and 1.05, respectively; all pHolm < 0.001) and for 7 vs. 14 days (MD 0.31; pHolm < 0.001). By contrast, the difference between 7 and 90 days was not statistically significant after multiplicity adjustment (MD 0.12; pHolm = 0.164), while the small contrast between 14 and 90 days reached significance (MD −0.18; pHolm = 0.020). Estimates are marginal means averaged over materials.

### 3.2. Color Difference (ΔE) over Time

**Figure 4:**
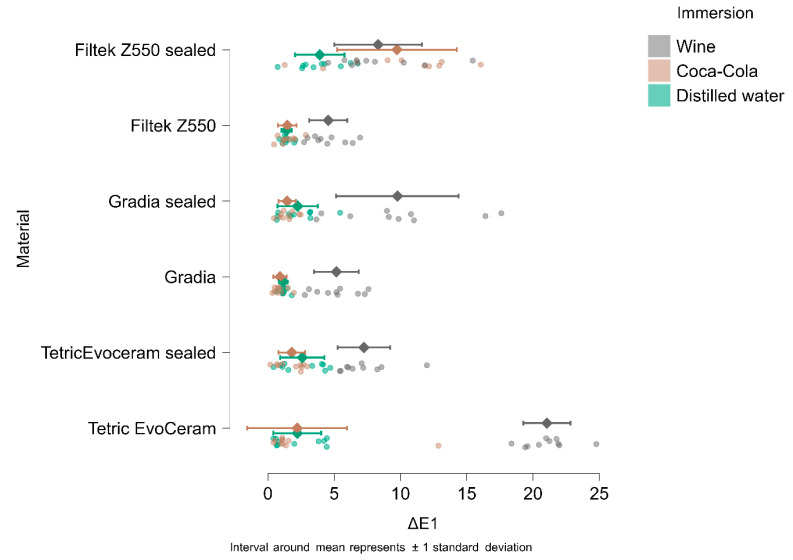
ΔE1 (ΔE baseline vs. 1 day of immersion) in wine, Coca-Cola, and distilled water.

**Figure 5:**
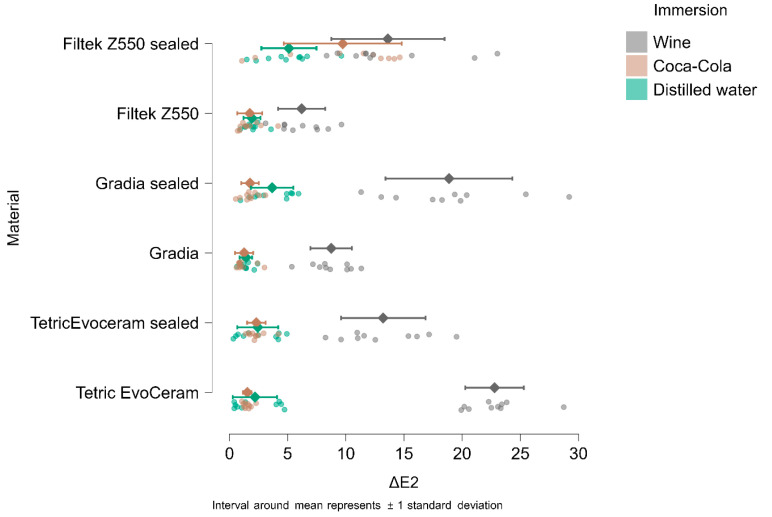
ΔE2 (ΔE baseline vs. 7 days of immersion) in wine, Coca-Cola, and distilled water.

**Figure 6:**
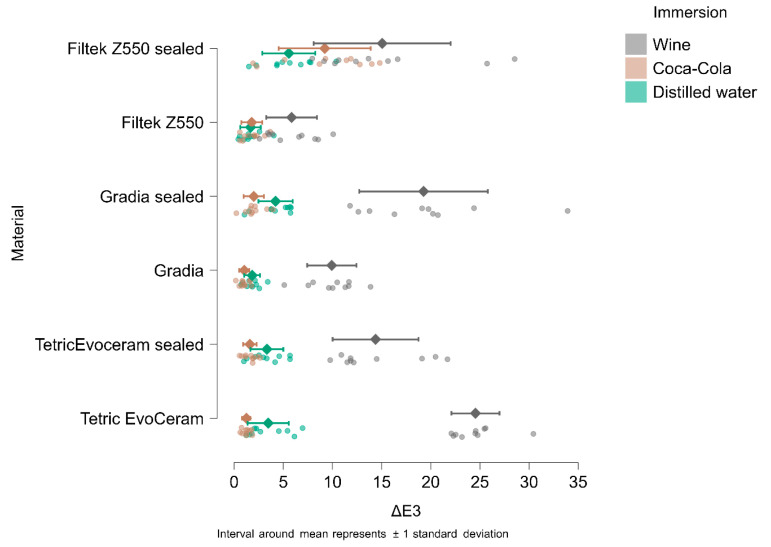
ΔE3 (ΔE baseline vs. 14 days of immersion) in wine, Coca-Cola, and distilled water.

**Figure 7:**
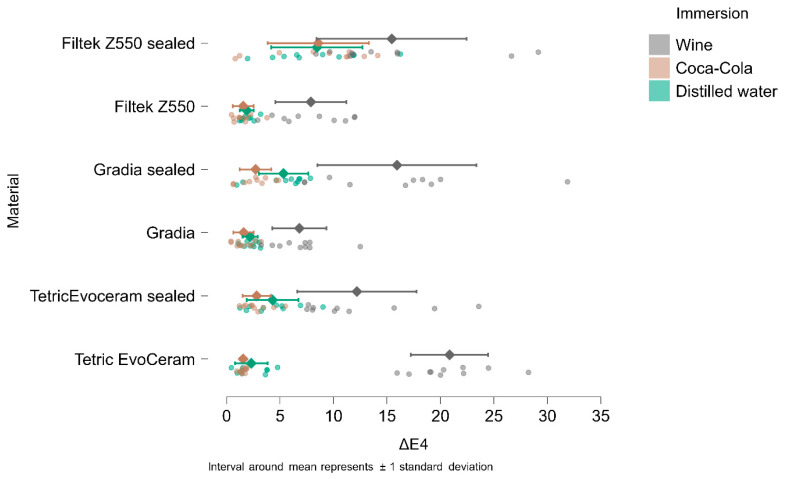
ΔE4 (ΔE baseline vs. 90 days of immersion) in wine, Coca-Cola, and distilled water.

In Figure 4, Figure 5, Figure 6 and Figure 7, the means and standard deviations are illustrated for Tetric Evo Ceram, Gradia, and Filtek with the two surface treatments (finished vs. sealed), the three immersion media (distilled water, Coca-Cola, and wine) and across the four time points.

After 1 day of immersion, the highest color change (ΔE1) was recorded for unsealed Tetric EvoCeram in wine (21.05), while the lowest color change was observed for unsealed Gradia in Coca-Cola (0.90).

After 7 days of immersion, the highest color change (ΔE2) was recorded for unsealed Tetric EvoCeram immersed in wine (22.78), and the lowest color change was observed for unsealed Gradia in Coca-Cola (1.25).

After 14 days of immersion, the highest color change (ΔE3) was recorded for unsealed Tetric EvoCeram immersed in wine (24,55), and the lowest color change was observed for unsealed Gradia in Coca-Cola (1.03).

After 90 days of immersion, the highest color change (ΔE4) was recorded for unsealed Tetric EvoCeram immersed in wine (20.84), and the lowest color change was observed for unsealed Filtek in Coca-Cola (1.55).

Additionally, an unpaired Student’s *t*-test was used to check for statistically significant differences between the sealed and unsealed surface treatments at all four time points and in all three different immersion media (Supplementary File S1 Tables S1–S3).

At day 1, the mean ΔE was 4.84, increasing to 6.59 at day 7 and peaking at 7.00 at day 14, which was followed by a slight reduction to 6.80 at day 90 (Table 4). Frequencies on the perceptibility scale indicated a clear temporal accumulation of discoloration: whereas 17.8% of samples showed no visible difference at day 1, this proportion declined to only 7.2% at day 90. Conversely, the “completely different shade” category rose from 27.2% at day 1 to 41.1% by day 90 (Table 4).

In short, the results show a clear trend over time: as ΔE increases from ΔE1 to ΔE4, the visibility of discoloration steadily grows. Specimens gradually shift from “not noticeable” or “only visible to an experienced observer” to “clearly different,” and by ΔE4, many reach the “completely different shade” category.

**Figure 8:**
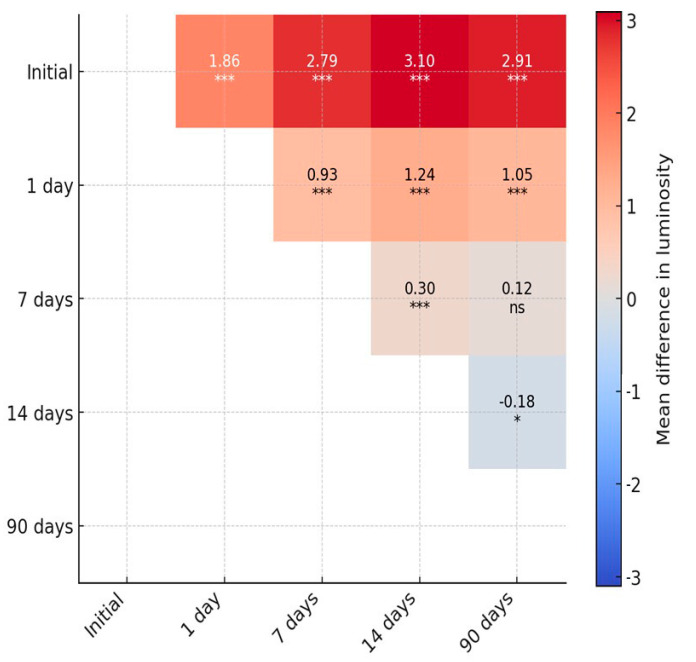
Heatmap of pairwise post hoc comparisons for luminosity. Cells show the mean difference in luminosity between conditions (row–column) together with Holm-adjusted significance (*** *p* ≤ 0.001, * *p* ≤ 0.05, ns non-significant). Only the upper triangle is displayed to avoid redundancy.

Post hoc pairwise contrasts confirmed that color change (ΔE*ab vs. baseline) increased from day 1 to day 7, 14, and 90 (MD −1.75 [95% CI −2.36 to −1.17], −2.16 [−2.84 to 1.47], and −1.96 [−2.60 to −1.31], respectively; all *p* < 0.001). Effects were small (Cohen’s d −0.28 [95% CI −0.38 to −0.18] to −0.35 [−0.47 to −0.23]). A modest additional rise occurred from day 7 to 14 (MD −0.42 [−0.68 to −0.15]; *p* < 0.001; d = −0.07 [−0.11 to −0.02]), after which values plateaued: day 7 vs. 90 and day 14 vs. 90 were not significant after multiplicity adjustment (*p* = 0.402 for both). Estimates are marginal means averaged over materials. This is also shown in Figure 8.

### 3.3. Material-Dependent Differences

The effect varies greatly depending on the material and whether it was sealed.

Filtek Z550 sealed performs worst overall—at ΔE1, 66.7% of specimens are already in the “two colors” category, and by ΔE4, 83.3% are “completely different shade.”

Gradia sealed follows, with 56.7% at ΔE4, and Tetric EvoCeram sealed, with 50% at ΔE4.

Unsealed variants are generally less affected by the end of the period (Tetric 33.3%, Filtek 26.7%, Gradia 23.3% “completely different shade” at ΔE4), though unsealed Tetric shows a higher proportion of severe cases from ΔE1 compared to unsealed Gradia or Filtek.

Overall, sealing does not provide consistent protection. For Filtek and Gradia, it seems to accelerate color change across the entire period, while for Tetric, the differences are smaller initially, but after 90 days, the sealed version still performs worse.

These trends are reported per material (aggregated across immersion media), highlighting both the increase in severity over time and the strong dependence on composite type.

Between-subjects comparisons highlighted that immersion in Coca-Cola and wine produced significantly higher ΔE values compared to distilled water (control). Coca-Cola immersion was associated with earlier perceptible differences, whereas wine immersion showed more variable but ultimately higher cumulative discoloration at later time points.

The mixed-model ANOVA for color difference relative to baseline (ΔE) showed a clear main effect of time, indicating that ΔE changed significantly across the four aging intervals (F(3.486) = 95.53, *p* < 0.001, η^2^ = 0.017). Time effects were further qualified by significant interactions with material (F(15,486) = 8.65, *p* < 0.001, η^2^ = 0.008), immersion medium (F(6.486) = 56.67, *p* < 0.001, η^2^ = 0.020) and their combined effect (time × material × immersion: F(30,486) = 5.66, *p* < 0.001, η^2^ = 0.010), demonstrating that the evolution of color change over time depended on both the restorative material and the storage solution. Between-subjects tests confirmed robust main effects of material (F(5.162) = 29.08, *p* < 0.001, η^2^ = 0.132) and immersion medium (F(2.162) = 264.76, *p* < 0.001, η^2^ = 0.479) together with a significant material × immersion interaction (F(10,162) = 17.79, *p* < 0.001, η^2^ = 0.161).

**Figure 9:**
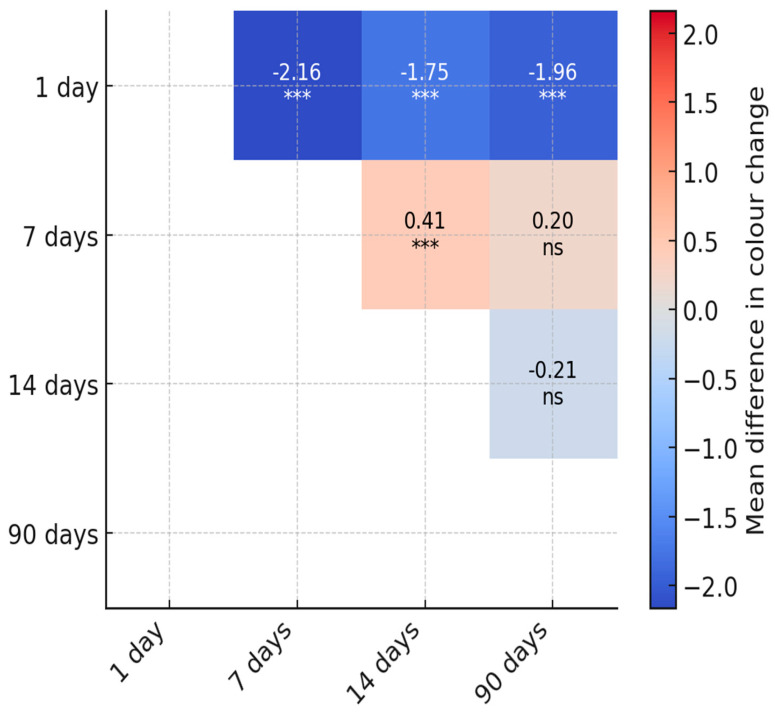
Heatmap of pairwise post hoc comparisons for color change (difference compared to initial value). Cells show the mean difference between conditions (row–column) together with Holm-adjusted significance (*** *p* ≤ 0.001, ns—non-significant). Only the upper triangle is displayed to avoid redundancy.

Thus, both the choice of composite and the aging solution substantially influenced the magnitude of color change, and the impact of the surface sealant differed across materials within each liquid. This is also shown in Figure 9.

Event rates were higher under the revised threshold definition (53 events, 127 censored), indicating that a considerable proportion of specimens exceeded the ΔE > 2 threshold within 90 days. Survival curves again showed clear separation by material. Tetric EvoCeram sealed and Gradia sealed demonstrated the most favorable profiles with restricted mean survival times of 90 days and median survival not reached by 90 days. Gradia also sustained high survival (RMST = 90 days; median = 90 days). In contrast, unsealed Tetric EvoCeram and Filtek Z550 showed earlier failures, with RMST values of 66.99 and 64.92 days, respectively, and median survival times of 90 days and 14 days. Filtek Z550 sealed showed the weakest resistance with a restricted mean survival time of 45.33 days and a median survival of only 7 days.

**Figure 10:**
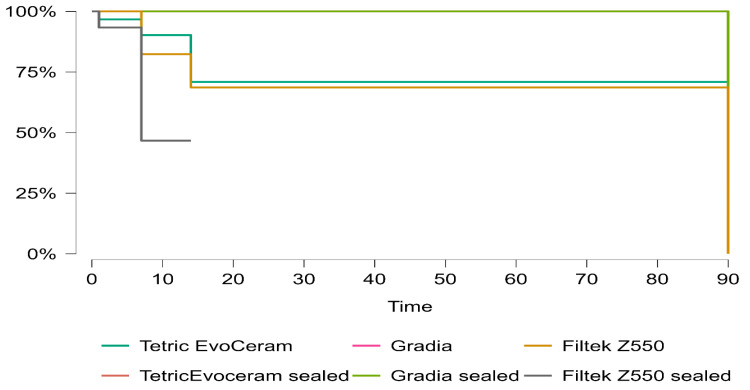
Kaplan–Meier survival curves illustrating the probability of maintaining ΔE ≤ 2 over 90 days stratified by material and sealing status. Sealed composites (particularly Tetric EvoCeram sealed and Gradia sealed) maintained the highest survival, while Filtek Z550 sealed exhibited the earliest threshold crossings (median survival = 7 days).

These results confirm a material-driven hierarchy of color stability with sealed composites (particularly Tetric EvoCeram sealed) maintaining the greatest resistance to perceptible discoloration (Figure 10).

### 3.4. Surface Roughness

The mean surface roughness (Ra) values for each composite, with and without the surface sealant, at all time points were analyzed individually. At baseline, Ra ranged from 0.150 to 0.364 µm with sealed specimens showing lower roughness than their unsealed counterparts for all materials (Tetric EvoCeram 0.261 vs. 0.203 µm, Gradia 0.276 vs. 0.191 µm, Filtek Z550 0.364 vs. 0.150 µm). After 1 day of immersion, Ra changed only slightly for most groups (0.254–0.339 µm for Tetric EvoCeram, 0.184–0.310 µm for Gradia), whereas Filtek Z550 sealed showed a transient increase (0.594 µm). At 7 and 14 days, roughness values remained in a similar range, and the sealed groups consistently presented smoother surfaces than the corresponding unsealed composites (e.g., at 14 days: Tetric EvoCeram 0.280 vs. 0.196 µm, Gradia 0.304 vs. 0.194 µm, Filtek Z550 0.346 vs. 0.271 µm). After 90 days, Ra increased markedly in all groups (1.638–1.989 µm for unsealed vs. 1.165–1.263 µm for sealed specimens), but sealing still reduced roughness relative to unsealed surfaces for each composite. Overall, Filtek Z550 tended to show the highest Ra values, whereas Tetric EvoCeram sealed exhibited the lowest, indicating that both composite type and surface coating influenced the evolution of surface topography over time.

For the analyses below, we used the following tests (α = 0.05): a one-sample *t*-test on dRa for each combination of material × treatment × immersion at each time point (1, 7, 14 and 90 days) to test whether the surface became rougher relative to baseline (dRa > 0). We report the exact *p*-value for each test.

Welch’s *t*-tests were used to compare the Ra values between different immersion media (water vs. Cola, water vs. wine, Cola vs. wine) within each material × treatment × time combination. Only comparisons with *p* < 0.05 are listed.

Welch’s *t*-tests were used to compare unsealed vs. sealed surfaces for each material, immersion medium and time point. The direction (which variant is rougher) and *p*-value are reported for all significant results.

The tables available in Supplementary File S1 (Tables S4–S6) list the *p*-values from the one-sample *t*-tests on dRa for each combination of material, surface variant and immersion medium. Columns correspond to time points relative to baseline. Small *p*-values (*p* < 0.05) indicate a significant increase in roughness.

For Tetric EvoCeram and Gradia, only the 90-day measurements show significant increases in roughness across all media and treatments (*p* ≤ 0.011), indicating that these materials remain stable during the first two weeks of immersion but become rougher after prolonged exposure.

Gradia sealed in water exhibits a slight but significant increase at 1 day (*p* = 0.043), although the mean change is very small (≈0.06 µm). The effect does not persist at later time points.

For Filtek Z550, the unsealed surfaces behave similarly to Tetric and Gradia, becoming significantly rougher only after 90 days (*p* ≤ 10^−5^). However, Filtek sealed in water shows a highly significant increase in roughness as early as 1 day (*p* ≈ 5 × 10^−5^) that continues at 7, 14 and 90 days.

Sealed Filtek in Coca-Cola and wine also become significantly rougher at 90 days (*p* < 0.001) but not at earlier time points.

**Figure 11:**
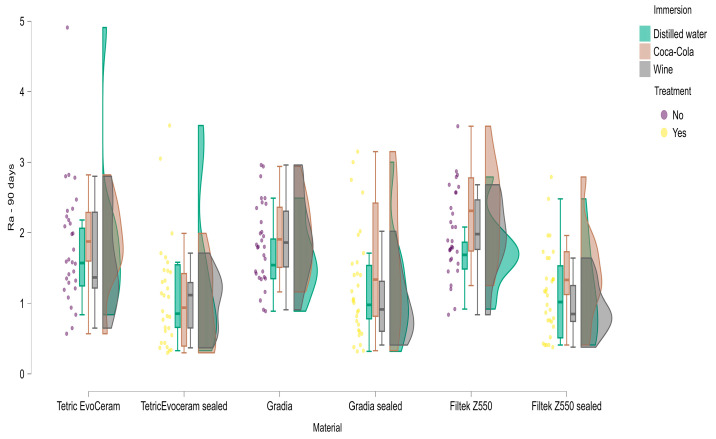
Distribution of surface roughness (Ra, µm) after 90 days of immersion by composite material, surface treatment (sealed vs. unsealed) and immersion medium (distilled water, Coca-Cola, red wine). Violin plots show the full distribution of Ra for each group; central boxplots indicate median and interquartile range with whiskers representing the range of non-outlier observations. Colored points represent individual specimens. Higher Ra values correspond to rougher surfaces.

These trends are illustrated in Figure 11, which displays the distribution of Ra values at 90 days for each composite stratified by surface treatment and immersion medium. The violin–boxplots highlight that for all three materials, unsealed specimens generally exhibit higher and more dispersed roughness values than their sealed counterparts with particularly elevated Ra for Filtek Z550 in all solutions. In contrast, sealed specimens cluster at lower Ra values, especially in distilled water, confirming the protective effect of the surface coating against long-term roughening in both neutral and acidic media.

Table S7 (provided the Supplementary File S1) summarizes the tests for dRa: each material × treatment × medium combination is listed, and the columns mark the time points at which the roughness increases significantly compared to the initial value. The symbol “↑” means that Ra increased significantly (*p* < 0.05; “–“ means it did not change significantly (*p* > 0.05).

For Tetric and Gradia (both Unsealed and sealed), roughness does not change significantly in the first 14 days, but it increases significantly at 90 days regardless of the immersion medium.

Sealed Gradia in water shows a small but significant increase as early as 1 day; however, the effect is small (dRa ≈ 0.06 μm) and is not observed again at 7 and 14 days.

Unsealed Filtek Z550 behaves similarly: the surface remains stable until 90 days when the roughness increases sharply (dRa ≈ 1.36–1.86 μm).

Sealed Filtek Z550 is an exception: in distilled water, it increases significantly as early as 1 day (dRa ≈ 1.10 μm) and continues to be rougher at 7, 14, and 90 days. In Coca-Cola and wine, the sealed variant only becomes rougher at 90 days.

Welch’s *t*-tests were performed to compare unsealed (unsealed) and sealed variants for each material, medium and time point. Table S8 (included in the Supplementary File S1) lists combinations where a significant difference (*p* < 0.05) was observed with the direction indicated.

Long-term immersion is the primary driver of increased surface roughness. Tetric EvoCeram and Gradia (both unsealed and sealed) show no significant roughening until the 90-day mark. Filtek Z550, particularly when sealed and immersed in water, roughens rapidly within the first day and continues to roughen thereafter.

Immersion medium has a relatively minor influence. A handful of significant differences appear: for example, Tetric sealed becomes rougher in wine than in water at 7 days, and sealed Filtek in water is rougher than the same material in Coca-Cola or wine at 1 and 14 days, but most comparisons yield *p* > 0.05.

Surface sealing generally reduces roughness. Unsealed surfaces tend to become significantly rougher than sealed ones as immersion time increases notably for Gradia and Filtek. An exception is Filtek sealed in water at 1 day, where the sealant layer appears to degrade or swell, temporarily making the sealed surface rougher than its unsealed counterpart.

The *p*-values reported above provide a complete picture of statistical significance across all comparisons performed.

### 3.5. Roughness and Correlation with Discoloration

Initial roughness values (Ra, Rz) were significantly altered by surface treatment, and their correlation with ΔE was modest. At day 1, no significant association was observed (r = −0.082 to −0.028, ns). By day 7, a weak but significant correlation emerged between Rz and ΔE (r = −0.211, *p* = 0.004), persisting with similar magnitude at days 14 and 90 (Figure 8).

Although statistically significant, effect sizes were small, suggesting that while surface roughness contributes to discoloration, immersion medium and material type remain the dominant determinants.

### 3.6. Predictors of Clinically Relevant Color Change

The multivariable model predicting the maintenance of barely perceptible discoloration at 90 days fit better than the intercept-only model (Δχ^2^ = 26.871, df = 8, *p* < 0.001). Model fit indices indicated modest–moderate explanatory power (McFadden R^2^ = 0.288; Nagelkerke R^2^ = 0.343; Tjur R^2^ = 0.166; Cox–Snell R^2^ = 0.139), and discrimination was good. After adjustment, no individual material or immersion term reached statistical significance on Wald tests (e.g., Gradia: β = −0.126, *p* = 0.892; Coca-Cola vs. water: *p* = 0.149; wine vs. water: *p* = 0.994), and sealing terms likewise did not attain significance (Table 5).

**Figure 12:**
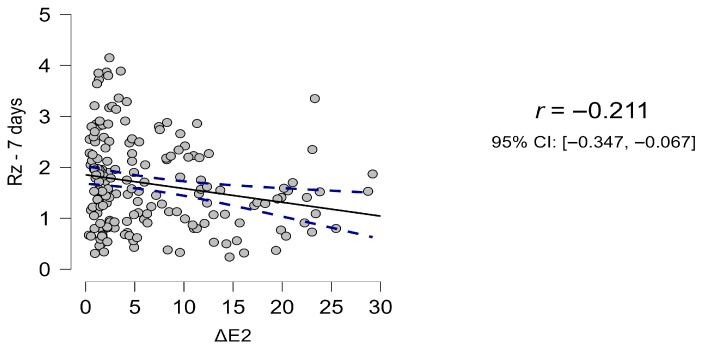
Scatterplot of color change versus surface roughness. The least-squares fit with 95% confidence band indicates a weak but statistically significant inverse association (r = −0.211; 95% CI −0.347 to −0.067; *p* = 0.004), explaining ~4.5% of the variability (R^2^ ≈ 0.045). The negative slope suggests that specimens with greater discoloration tended to show slightly lower Rz values.

**Figure 13:**
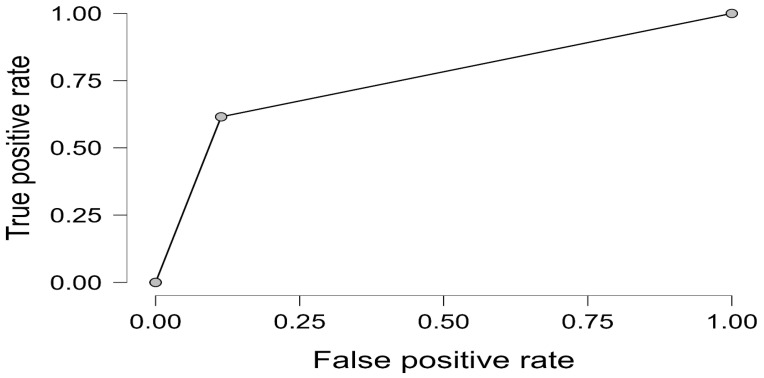
ROC curve for the logistic regression model predicting clinically relevant discoloration at 90 days. The model demonstrated good discriminatory ability, with an AUC of 0.877, indicating reliable prediction of perceptible color change.

Although none of the individual material or immersion predictors reached statistical significance at the Wald test level (all *p* > 0.1), the overall model performance was robust with an area under the ROC curve (AUC) of 0.877 (Figure 12 and Figure 13). This confirms the good discriminatory capacity of the model for differentiating specimens likely to resist clinically perceptible discoloration over 90 days.

Collectively, the findings demonstrate that discoloration was primarily driven by time and material with immersion medium acting as an amplifier. ΔE increased steeply from day 1 to day 14 and then stabilized with a progressive shift of specimens into higher perceptibility bands; Coca-Cola and wine produced greater changes than water. Sealed Tetric EvoCeram and sealed Gradia exhibited the most robust stability, maintaining high survival with few events by 90 days, whereas unsealed Tetric EvoCeram and Filtek Z550 failed earlier, and sealed Filtek Z550 showed the weakest resistance (median survival = 7 days). Surface sealing consistently reduced the risk of early perceptible change. Surface roughness correlated only weakly with ΔE, confirming it as a minor determinant compared with material and immersion medium. In multivariable modelling, no single factor reached significance, but the model as a whole discriminated well (AUC = 0.877), reinforcing that overall, material choice and sealing status remain central to long-term color stability.

## 4. Discussion

In everyday practice, the assessment of color changes over time is often used to determine the effectiveness of dental resin composites [38].

Coloring is classified into two types: internal and external. Changes in the resin matrix, filler, loading, particle size distributions, and photoinitiator type all contribute to internal color change [39]. On the other hand, polishing is an easy way to remove external discoloration that may be caused by poor dental hygiene, smoking, diet and the retention of stains that are water-soluble throughout the resin matrix [40,41]. Unlike the external sources, internal color modifications cannot be reversed [42].

Resin-based dental products like BisCover and Permaseal are surface sealants (resin-based dental materials with low viscosity) that have been developed to better preserve the resin composite’s esthetic appearance and surface smoothness characteristics by filling the micro-defects within the composite’s surface structure [31,43]. Surface sealant contains monomers such as Bis-GMA (bisphenol glycidyl dimethacrylate), UDMA (urethane dimethacrylate), TEGDMA (triethylene glycol dimethacrylate), and THFMA (tetrahydrofurfuryl methacrylate) [33].

On the other hand, due to financial constraints, clinical efficiency considerations, and the fact that some studies have reported similar results when using an adhesive versus a surface sealant for preserving surface roughness, the adhesive system was tested as a surface sealant in this study [29].

Due to its mild pH (2.7), Scotchbond Universal is less vulnerable to hydrolytic breakdown, resulting in better adhesive performance [44]. Moreover, some studies stated that 10-methacryloyloxydecyl dihydrogen phosphate (10-MDP), an adhesive monomer of Scotchbond Universal’s structure, is capable of forming chemical bonding with dental tissue, metals, and even nanofilled zirconia particles in resin composites [44,45,46,47]. One disadvantage of the adhesive system is that the presence of solvents can reslt in incomplete photopolymerization [36].

Regarding the changes recorded over the four time points, when converted to clinical interpretation, the color difference increased from ΔE1 to ΔE4, reaching the category of “completely different shade” at the final measurement.

Regarding the immersion media, in this research immersion in Coca-Cola and wine produced significantly higher ΔE values in comparison to distilled water (control). The determinant factors for choosing red wine as a staining agent were its low pH, the presence of numerous pigment-producing molecules, and its high alcohol content [48,49]. These factors may be responsible for the pronounced color change observed in resin composites in previous research [48,50] In this research, red wine demonstrated the highest pigmentation potential. The observation drawn from our current study that Coca-Cola (known to be highly acidic and to contain increased pigment concentrations) shows lower staining ability compared to red wine has also been reported in other research. This may be attributed to the low polarity of these staining agents [49].

In this work, based on the statistical analysis, the following conclusion can be drawn: color stability is strongly related to the material with sealed composites (particularly Tetric EvoCeram sealed and Gradia sealed) maintaining the highest resistance to noticeable color change.

The beneficial use of the surface sealing was indicated in another research, highlighting that it might enhance the stain resistance of composite resin materials [26]. In this study, E values gradually rose from ΔE1 to ΔE4. By ΔE4, the samples had changed from “not noticeable” to “clearly different” to “completely different shade”. Also, over 90 days, Tetric EvoCeram sealed and Gradia sealed were the most resistant to visible color changes.

The outcomes were greatly affected by the material and the sealing, but sealing did not always prevent color change. This is supported by other research, which indicated that the color maintenance of microhybrid composite resins was negatively affected by the application of surface sealants [51].

In this study, over time, sealing makes the surface less rough. Some studies have proposed the use of surface sealants to fill the pores, for better wear resistance, for the preservation of surface smoothness, and for the promotion of structural integrity, thus, enhancing the surface properties [15,52]. Ninety days of immersion is the main cause of surface roughening in this study, as Tetric EvoCeram and Gradia maintained surfaces that were smooth for up to 14 days.

Moreover, when it comes to the roughening process, statistics showed that the kind of material and the surface treatment were more relevant than the type of immersion media. Some authors have proposed that a particularly developed penetrating sealant may be advantageous to increase the degree of penetration into the surface irregularities, helping the maintenance of moisture and sealing surface defects [30,53].

Another research study examined the roughness of a resin-based composite post-polishing and after the application of surface sealants, concluding that similar roughness levels may be attained using either method [15].

In one study, where the adhesive was compared with surface sealants, the adhesive systems showed inferior color stability than the surface sealants and the unsealed resin composites with the exception of Single Bond [32,54].

In this investigation, the immersion periods were chosen based on the assumption that one month of in vitro staining simulates approximately one year of beverage consumption, as reported by some researchers [55,56,57,58,59,60]. Consequently, the time periods would correspond as follows: 1 day–1 month, 7 days–7 months, 14 days–1 year, 90 days–seven and a half years.

Also, the temperatures were chosen to reflect how the drinks are normally enjoyed: 37 °C, which is the normal human body temperature for water, and 10 °C, the temperature in a refrigerator where Coca-Cola and red wine are usually stored before being consumed. In addition, the chosen temperature of 37 °C and continuous immersion in coloring beverages were selected because these conditions are also believed by some researchers to replicate the oral environment [52].

Regarding the material-dependent results, these results could also be attributed to their compositions. The organic matrix of a resin composite is composed of monomers (such as Bis-GMA, UDMA or TEGDMA) and initiators, while the inorganic phase consists of filler particles (such as zirconia or glass ceramics). These monomers are directly related to water sorption; for example, UDMA has been shown to be less soluble in water than Bis-GMA, which is known to be the most inclined to water absorption [61,62]. The hydrolytic effect of water lies in its ability to plasticize, and therefore weaken, the matrix–filler bond by interrupting the silane–filler connection [10]. Their performance could be attributed to their Bis-GMA content.

Another important factor related to their internal structure, water sorption properties, and degradation of optical/surface characteristics is the type and amount of fillers. The literature highlights that resin composites with a lower tendency to water degradation are those with a highly filled matrix and less available space for water molecules [61,62].

In this study, Filtek Z550 had the highest degree of filler, but in contradiction with its expected behavior, it performed the worst in terms of maintaining its optical properties. This is in accordance with Nasim et al.’s findings, which showed that resin composites with nanofillers exhibited greater color change when exposed to various liquids compared to microhybrid resins [63].

Tetric EvoCeram sealed, followed by Gradia sealed, performed the best regarding their behavior over the 90 days of immersion, as shown by the survival curve. This result could be attributed to their composition.

Conversely, the first null hypothesis was rejected, as there were differences between the sealed/unsealed materials. The second and third null hypothesis was rejected, as there were significant differences regarding the storage media and the time points regarding the color change and the surface roughness. The forth null hypothesis was partially approved, as there was a weak correlation between the surface roughness and color change.

Future perspectives are outlined below:

(1) The testing medium—other immersion media should be considered in future studies, such as artificial saliva, coffee, or curry;
(2) The effect of brushing or simulated oral attrition was not tested;
(3) Other polishing methods should be considered in future investigations;
(4) Other aging methods, such as thermocycling, were not employed;
(5) The effect of different temperatures was not tested;
(6) Results are based on in vitro simulations and may not directly translate to long-term intraoral performance;
(7) Other materials or categories of materials should be taken into account.

Consequently, more research is required to assess the real efficacy of various resin composites in the oral cavity.

## 5. Conclusions

Within the limitations of this study, it can be concluded that sealing may help preserve both color stability and surface roughness. Regarding surface roughness, the sealed surfaces showed the best performance. A clinical approach is recommended to complete these in vitro findings, taking into account the clinician’s perspective on cost-effectiveness and time required, as well as the patient’s long-term follow-up in maintaining the outcomes of sealed (using different surface sealings)/finished (using different finishing procedures) of dental direct restorations in the mouth.

## Figures and Tables

**Table 1:** Direct resin composites used in this study.

Material	Manufacturer	Organic Matrix	Inorganic Filler	Filler Content (%)
Gradia Direct Anterior A2	GC Corporation, Tokyo, Japan	UDMA, Bis-EMA, dimethacrylates, trimethacrylates	Barium glass, silica, prepolymerized resin fillers	69
Tetric Evo Ceram A2	Ivoclar Vivadent, Liechtenstein	Bis-GMA, Bis-EMA, TEGDMA	Prepolymerized fillers, silica	75
Filtek Z550 A2	3M ESPE, USA	Bis-GMA, UDMA, TEGDMA, BIS-EMA, PEGDMA	Zirconia/silica nanoparticles	78.5
Scotchbond Universal (US)	3M ORAL CARE ST. PAUL MN, USA		Ethyl alcohol 25% to 35%, bisphenol a diglycidyl ether dimethacrylate (bisgma) 10% to 20%, silane-treated silica none 10% to 20%, 2-hydroxyethyl methacrylate (hema) 5–15%, glycerol 1,3-dimethacrylate 1830-78-0 5% to 10%, copolymer of acrylic and itaconic acids 5% to 10%, water < 5%, diurethane dimethacrylate (udma) < 5%, diphenyliodonium hexafluorophosphate < 1%, ethyl 4-dimethyl aminobenzoate (edmab) < 1%	

**Table 2:** Repeated-measures ANOVA for luminosity across immersion media and materials’ within-subjects effects.

Cases	Sum of Squares	df	Mean Square	F	*p*	η^2^
Luminosity	1186.2	4	296.548	278.74	<0.001	0.08
Luminosity × Immersion	2185.3	8	273.163	256.76	<0.001	0.15
Luminosity × Material	338.7	20	16.935	15.92	<0.001	0.02
Luminosity × Immersion × Material	691.6	40	17.290	16.25	<0.001	0.04
Residuals	689.4	648	1.064			

**Table 3:** Repeated-measures ANOVA for luminosity across immersion media and materials’ between-subjects effects.

Cases	Sum of Squares	df	Mean Square	F	*p*	η^2^
Immersion	3963.3	2	1981.651	286.09	<0.001	0.28
Material	3014.9	5	602.989	87.05	<0.001	0.21
Immersion Material	904.9	10	90.492	13.06	<0.001	0.06
Residuals	1122.1	162	6.927			

**Table 4:** Distribution of ΔE perceptibility scores across evaluation scales (ΔE1–ΔE4).

ΔE Category	ΔE1 n (%)	ΔE2 n (%)	ΔE3 n (%)	ΔE4 n (%)
Difference not visible	32 (17.8)	22 (12.2)	20 (11.1)	13 (7.2)
Difference barely visible	45 (25.0)	41 (22.8)	41 (22.8)	40 (22.2)
Small visible difference	17 (9.4)	23 (12.8)	21 (11.7)	20 (11.1)
Clearly visible difference	37 (20.6)	29 (16.1)	31 (17.2)	33 (18.3)
Completely different shade	49 (27.2)	65 (36.1)	67 (37.2)	74 (41.1)
Total	180 (100)	180 (100)	180 (100)	180 (100)

**Table 5:** Logistic regression models predicting clinically discoloration at 90 days. Model M_0_ = intercept-only; Model M_1_ = full model including immersion medium, material, and surface treatment. The full model showed significantly improved fit (Δχ^2^ = 26.871, df = 8, *p* < 0.001) with higher explanatory power across multiple pseudo-R^2^ indices.

Model	Deviance	AIC	BIC	df	*p*	McFadden R^2^	Nagelkerke R^2^	Tjur R^2^
M_0_	93.37	95.366	98.559	179	-	0.000	-	0.000
M_1_	66.49	84.495	113.231	171	<0.001	0.288	0.343	0.166

## Data Availability

The original contributions presented in this study are included in the article/Supplementary Materials. Further inquiries can be directed to the corresponding author.

## Supplementary Materials

The following supporting information can be downloaded at https://www.mdpi.com/article/10.3390/ma18245543/s1, Table S1: *p*-values for pairwise comparisons of colour difference (ΔE) between unsealed and sealed surface treatments for T (Tetric), Ts (Tetric sealed), G (Gradia), Gs (Gradia sealed), F (Filtek), and Fs (Filtek sealed) at four time points in distilled water; Table S2: *p*-values for pairwise comparisons of colour difference (ΔE) between unsealed and sealed surface treatments for T (Tetric), Ts (Tetric sealed), G (Gradia), Gs (Gradia sealed), F (Filtek), and Fs (Filtek sealed) at four time points in Coca-Cola; Table S3: *p*-values for pairwise comparisons of colour difference (ΔE) between unsealed and sealed surface treatments for T (Tetric), Ts (Tetric sealed), G (Gradia), Gs (Gradia sealed), F (Filtek), and Fs (Filtek sealed) at four time points in red wine; Table S4: *p*-value for Tetric EvoCeram sealed vs unsealed in the three imersion media, at the four different time points; Table S5: *p*-value for Gradia sealed vs unsealed in the three imersion media, at the four different time point; Table S6: *p*-value for Filtek sealed vs unsealed in the three imersion media, at the four different time points; Table S7: Summary of statistically significant changes in surface roughness (Ra) for Tetric EvoCeram, Gradia and Filtek Z550 composites, with and without surface sealant, after immersion in the three solutions (distilled water, Coca-Cola and red wine) at four time points (1, 7, 14 and 90 days); Table S8: “Unsealed↑” indicates that the Unsealed surface is rougher; “Sealed↑” indicates that the sealed surface is rougher.”, and “–” means no difference.
