# Supplementary material for: Impact of Surface Sealing on Color Stability and Surface Roughness of Conventional Dental Resin Composites
Source: Materials (Basel). 2025 Dec 10;18(24):5543. doi: 10.3390/ma18245543 (PMC12734704; doi:10.3390/ma18245543)
Supplement: Supplementary file 1 [file materials-18-05543-s001.zip › materials-3989335-supplementary File S1.pdf]

*Article*

# Impact of Surface Sealing on Color Stability and Surface Roughness of Conventional Dental Resin Composites

**Georgiana Osiceanu [1,*], Sebastian Ciurescu [2] and Liliana Porojan [3]**

[1] Department of Dental Prostheses Technology (Dental Technology), Center for Advanced Technologies in Dental Prosthodontics, Faculty of Dental Medicine, Doctoral School, Victor Babes University of Medicine and Pharmacy Timisoara, Eftimie Murgu Square No. 2, 300041 Timisoara, Romania
[2] Doctoral School, Victor Babes University of Medicine and Pharmacy Timisoara, Eftimie Murgu Square No. 2, 300041 Timisoara, Romania
[3] Department of Dental Prostheses Technology (Dental Technology), Center for Advanced Technologies in Dental Prosthodontics, Faculty of Dental Medicine, Victor Babes University of Medicine and Pharmacy Timisoara, Eftimie Murgu Square No. 2, 300041 Timisoara, Romania
* Correspondence: georgiana.osiceanu@umft.ro

**Table S1.** P-values for pairwise comparisons of colour difference ($\Delta E$) between unsealed and sealed surface treatments for T (Tetric), Ts (Tetric sealed), G (Gradia), Gs (Gradia sealed), F (Filtek), and Fs (Filtek sealed) at four time points in distilled water. $\Delta E1$ = colour difference between baseline and 1 day of immersion; $\Delta E2$ = colour difference between baseline and 7 days; $\Delta E3$ = colour difference between baseline and 14 days; $\Delta E4$ = colour difference between baseline and 90 days.

| **Distilled water** | **p-value** | | | | |
|---|---|---|---|---|---|
| | **T-Ts** | **G-Gs** | **F-Fs** | **T-G-F** | **Ts-Gs-Fs** |
| **Δ E1** | 0.529 | 0.121 | 0.002 | 0.092 | 0.084 |
| **Δ E2** | 0.791 | 0.03 | 0.002 | 0.365 | 0.02 |
| **Δ E3** | 0.971 | 0.007 | <0.001 | 0.016 | 0.07 |
| **Δ E4** | 0.063 | 0.015 | <0.001 | 0.629 | 0.016 |

**Table S2.** P-values for pairwise comparisons of colour difference ($\Delta E$) between unsealed and sealed surface treatments for T (Tetric), Ts (Tetric sealed), G (Gradia), Gs (Gradia sealed), F (Filtek), and Fs (Filtek sealed) at four time points in Coca-Cola. $\Delta E1$ = colour difference between baseline and 1 day of immersion; $\Delta E2$ = colour difference between baseline and 7 days; $\Delta E3$ = colour difference between baseline and 14 days; $\Delta E4$ = colour difference between baseline and 90 days. Statistically significant results ($p < 0.05$) are shown in bold.

| **Coca-Cola** | **p-value** | | | | |
|---|---|---|---|---|---|
| | **T-Ts** | **G-Gs** | **F-Fs** | **T-G-F** | **Ts-Gs-Fs** |
| **Δ E1** | 0.307 | 0.054 | 0.002 | 0.4363 | <0.001 |
| **Δ E2** | 0.05 | 0.104 | 0.002 | 0.401 | <0.001 |
| **Δ E3** | 0.247 | 0.009 | <0.001 | 0.076 | <0.001 |
| **Δ E4** | 0.011 | 0.063 | 0.004 | 0.989 | <0.001 |

**Table S3.** P-values for pairwise comparisons of colour difference (ΔE) between unsealed and sealed surface treatments for T (Tetric), Ts (Tetric sealed), G (Gradia), Gs (Gradia sealed), F (Filtek), and Fs (Filtek sealed) at four time points in red wine. ΔE1 = colour difference between baseline and 1 day of immersion; ΔE2 = colour difference between baseline and 7 days; ΔE3 = colour difference between baseline and 14 days; ΔE4 = colour difference between baseline and 90 days. Statistically significant results ($p < 0.05$) are shown in bold.

| **Red wine** | **p-value** | | | | |
|---|---|---|---|---|---|
| | **T-Ts** | **G-Gs** | **F-Fs** | **T-G-F** | **Ts-Gs-Fs** |
| **Δ E1** | <0.001 | 0.015 | 0.002 | <0.001 | 0.279 |
| **Δ E2** | <0.001 | <0.001 | <0.001 | <0.001 | 0.02 |
| **Δ E3** | <0.001 | <0.001 | <0.001 | <0.001 | 0.168 |
| **Δ E4** | <0.001 | <0.001 | 0.007 | <0.001 | 0.412 |

**Table S4.** p- value for Tetric EvoCeram sealed vs unsealed in the three imersion media, at the four different time points.

| Tetric EvoCeram | p-value | | | |
|---|---|---|---|---|
| **Treatment × Medium** | **1 day** | **7 days** | **14 days** | **90 days** |
| Unsealed – Water | 0.599 | 0.514 | 0.459 | **0.002** |
| Unsealed – Cola | 0.574 | 0.784 | 0.911 | **<0.001** |
| Unsealed – Wine | 0.056 | 0.370 | 0.115 | **<0.001** |
| Sealed – Water | 0.573 | 0.146 | 0.658 | **0.011** |
| Sealed – Cola | 0.898 | 0.906 | 0.351 | **0.005** |
| Sealed – Wine | 0.299 | 0.146 | 0.332 | **<0.001** |

**Table S5.** p- value for Gradia sealed vs unsealed in the three imersion media, at the four different time point.

| Gradia | p-value | | | |
|---|---|---|---|---|
| **Treatment × Medium** | **1 day** | **7 days** | **14 days** | **90 days** |
| Unsealed – Water | 0.211 | 0.053 | 0.878 | **<0.001** |
| Unsealed – Cola | 0.305 | 0.127 | 0.922 | **<0.001** |
| Unsealed – Wine | 0.945 | 0.810 | 0.356 | **<0.001** |
| Sealed – Water | 0.043 | 0.991 | 0.395 | **0.002** |
| Sealed – Cola | 0.857 | 0.455 | 0.083 | **0.001** |

**Table S6.** p- value for Filtek sealed vs unsealed in the three imersion media, at the four different time points.

| Filtek Z550 | p-value | | | |
|---|---|---|---|---|
| **Treatment × Medium** | **1 day** | **7 days** | **14 days** | **90 days** |
| Unsealed – Water | 0.553 | 0.911 | 0.798 | **<0.001** |
| Unsealed – Cola | 0.536 | 0.856 | 0.264 | **<0.001** |
| Unsealed – Wine | 0.282 | 0.615 | 0.477 | **<0.001** |
| Sealed – Water | **<0.001** | **0.035** | **0.038** | **0.002** |
| Sealed – Cola | 0.103 | 0.226 | 0.243 | **<0.001** |
| Sealed – Wine | **0.051** | 0.069 | 0.900 | **<0.001** |

**Table S7.** Summary of statistically significant changes in surface roughness (Ra) for Tetric EvoCeram, Gradia and Filtek Z550 composites, with and without surface sealant, after immersion in the three solutions (distilled water, Coca-Cola and red wine) at four time points (1, 7, 14 and 90 days). An upward arrow ( ↑ ) indicates a statistically significant increase in Ra compared with baseline for that material/medium/treatment combination ($p < 0.05$, post-hoc tests with multiplicity adjustment); " – " indicates no significant change.

| Material | Treatment | Medium | 1 day | 7 days | 14 days | 90 days |
|---|---|---|---|---|---|---|
| Tetric EvoCeram | Unsealed | Water | – | – | – | ↑ |
| Tetric EvoCeram | Unsealed | Cola | – | – | – | ↑ |
| Tetric EvoCeram | Unsealed | Wine | – | – | – | ↑ |
| Tetric EvoCeram | Sealed | Water | – | – | – | ↑ |
| Tetric EvoCeram | Sealed | Cola | – | – | – | ↑ |
| Tetric EvoCeram | Sealed | Wine | – | – | – | ↑ |
| Gradia | Unsealed | Water | – | – | – | ↑ |
| Gradia | Unsealed | Cola | – | – | – | ↑ |
| Gradia | Unsealed | Wine | – | – | – | ↑ |
| Gradia | Sealed | Water | ↑ | – | – | ↑ |
| Gradia | Sealed | Cola | – | – | – | ↑ |
| Gradia | Sealed | Wine | – | – | – | ↑ |
| Filtek Z550 | Unsealed | Water | – | – | – | ↑ |
| Filtek Z550 | Unsealed | Cola | – | – | – | ↑ |
| Filtek Z550 | Unsealed | Wine | – | – | – | ↑ |
| Filtek Z550 | Sealed | Water | ↑ | ↑ | ↑ | ↑ |
| Filtek Z550 | Sealed | Cola | – | – | – | ↑ |
| Filtek Z550 | Sealed | Wine | – | – | – | ↑ |

**Table S8**. "Unsealed↑" indicates that the Unsealed surface is rougher; "Sealed↑" indicates that the sealed surface is rougher.", and "–" means no difference.

| Material | Medium | 0 days | 1 day | 7 days | 14 days | 90 days |
|---|---|---|---|---|---|---|
| **Tetric EvoCeram** | Water | – | – | Unsealed↑ | – | – |
| | Cola | – | – | – | – | Unsealed↑ |
| | Wine | – | – | – | – | Unsealed↑ |
| **Gradia** | Water | – | – | Unsealed↑ | – | – |
| | Cola | Unsealed ↑ | Unsealed↑ | Unsealed↑ | – | – |
| | Wine | – | Unsealed↑ | – | Unsealed↑ | Unsealed↑ |
| **Filtek Z550** | Water | Unsealed ↑ | Sealed↑ | – | – | – |

| | | | | | | |
|---|---|---|---|---|---|---|
| | Cola | Unsealed ↑ | – | Unsealed↑ | – | Unsealed↑ |
| | Wine | Unsealed ↑ | – | Unsealed↑ | Unsealed↑ | Unsealed↑ |
